# Hereditary Spherocytosis: Review of Presentation at Birth

**DOI:** 10.3390/children12091207

**Published:** 2025-09-10

**Authors:** Nadine-Stella Achenjang, Elizabeth Jadczak, Rita M. Ryan, Mary L. Nock

**Affiliations:** 1 Department of Pediatrics (Neonatology), University of Arizona-Tucson, Tucson, AZ 85721, USA; 2 Department of Pediatrics (Neonatology), Case Western Reserve University, UH Rainbow Babies and Children’s Hospital, Cleveland, OH 44106, USA

**Keywords:** red blood cell hemolysis, hyperbilirubinemia, G6PD deficiency, jaundice, eosin 5’ maleimide

## Abstract

**Background/Objectives**: We wished to raise awareness of Hereditary Spherocytosis (HS) as a potential cause of early and significant hemolytic newborn jaundice. **Methods**: We utilized three recent cases from our experience to discuss hyperbilirubinemia etiologies to be considered when a baby has hemolytic hyperbilirubinemia, including HS, and presented a review of the literature about this disorder including presentation and evaluation in the neonate. **Results**: We found that ABO hemolytic disease of the newborn (HDN) is often considered as the etiology for presumed hemolytic hyperbilirubinemia even when the direct antiglobulin test (DAT) is negative. When there is a mother-baby ABO mismatch and baby’sDAT is negative, another etiology should be sought. HS should be considered in these cases as the prevalence of HS is as frequent as 1 in 2000 in certain populations, it is the third most common hemolytic disorder after ABO isoimmunization and G6PD deficiency, and it is the most common cause of non-immune hemolytic hyperbilirubinemia in neonates with kernicterus. The indices to look for in the complete blood count that are suggestive for HS are MCHC > 36.5–37 g/dL, an MCHC:MCV ratio (HS Index) > 0.36, and increased RDW. The lack of spherocytes on the newborn peripheral blood smear, family history, initial anemia, and reticulocytosis do not eliminate the diagnosis of HS. **Conclusions**: HS is common and should be included in the differential diagnosis for hemolytic hyperbilirubinemia. Red blood cell indices can suggest the diagnosis of HS, and eosin 5’ maleimide (EMA) testing can be used to make the diagnosis. If DAT-negative ABO HDN is the leading diagnosis for hyperbilirbinemia, a different etiology should urgently be sought.

## 1. Introduction

Hyperbilirubinemia is commonly encountered in the newborn nursery and neonatal intensive care unit (NICU). As many as 80% of healthy, full-term newborns have elevated total serum bilirubin (TSB) levels, usually due to physiologic jaundice [1,2]. In term infants, this typically appears after 24 h of age and peaks on days of life (DOL) 2–5 with TSB of 5–6 mg/dL. In late preterm infants (34 0/7–36 6/7 weeks gestation at birth), a higher TSB peak of up to 10–12 mg/dL is seen at about the fifth day of life. Physiologic jaundice is benign and transient [2].

In contrast, pathologic jaundice, if not recognized and treated appropriately, can lead to acute bilirubin encephalopathy (BE) [1,2]. In rare cases, acute BE can progress to chronic BE or kernicterus with the sequelae of choreoathetoid cerebral palsy, dental enamel hypoplasia, upward gaze palsy, and sensorineural hearing loss [2]. Given these adverse effects, pathologic jaundice necessitates prompt recognition, treatment, and evaluation for an underlying cause. When total serum bilirubin (TSB) exceeds the 95th percentile hour-specific nomogram value within the first 24 h of life, this suggests pathologic jaundice from increased bilirubin production, and a hemolytic condition should be strongly considered [3]. Various laboratory indicators of hemolytic hyperbilirubinemia have been suggested (e.g., early elevated TSB with elevated reticulocyte count) with only end-tidal carbon monoxide (corrected for ambient carbon dioxide, ETCOc) suggested by experts as the gold standard for diagnosis of hemolysis in the neonate [3,4]. Nonetheless, identifying hemolysis as the cause of hyperbilirubinemia is critical as hemolysis is not only a risk for developing significant neonatal hyperbilirubinemia, but it is also a risk factor for neurotoxicity from hyperbilirubinemia [5].

When pathologic jaundice is encountered, infants should receive prompt treatment, as indicated by the 2022 American Academy of Pediatrics (AAP) Clinical Practice Guideline on hyperbilirubinemia, to decrease the risk of bilirubin toxicity and BE [5]. While phototherapy and exchange transfusion can treat pathologic hyperbilirubinemia from any etiology, recognizing hemolysis and identifying the cause of hemolysis are important for possible intravenous immunoglobulin (IVIG) infusion for alloimmune hemolytic disease of the newborn (HDN) [5] and for understanding the expected course of hyperbilirubinemia and anemia. In this report we share three cases of infants with early neonatal hyperbilirubinemia requiring phototherapy before 24 h of life ultimately diagnosed with hereditary spherocytosis (HS). None of the patients had a family history of HS, and the two patients with ABO mismatch with their mothers had negative direct antiglobulin tests (DATs).

## 2. Case Descriptions

### 2.1. Case I

A small for the gestational age female infant born at 2410 g at 37 weeks (w) 6 days (d) via spontaneous vaginal delivery was admitted to the NICU from the newborn nursery (NBN) due to persistent indirect hyperbilirubinemia. She was born to a 20-year-old Hispanic primigravid woman. Pregnancy was complicated by maternal hypothyroidism and anemia treated with levothyroxine and iron supplementation. Prenatal screens (PNS) were significant for group B streptococcus (GBS) positivity, with adequate intrapartum treatment. The mother’s blood type was O−, and she had a negative antibody screen; she received Rhogam during pregnancy.

**Figure 1:**
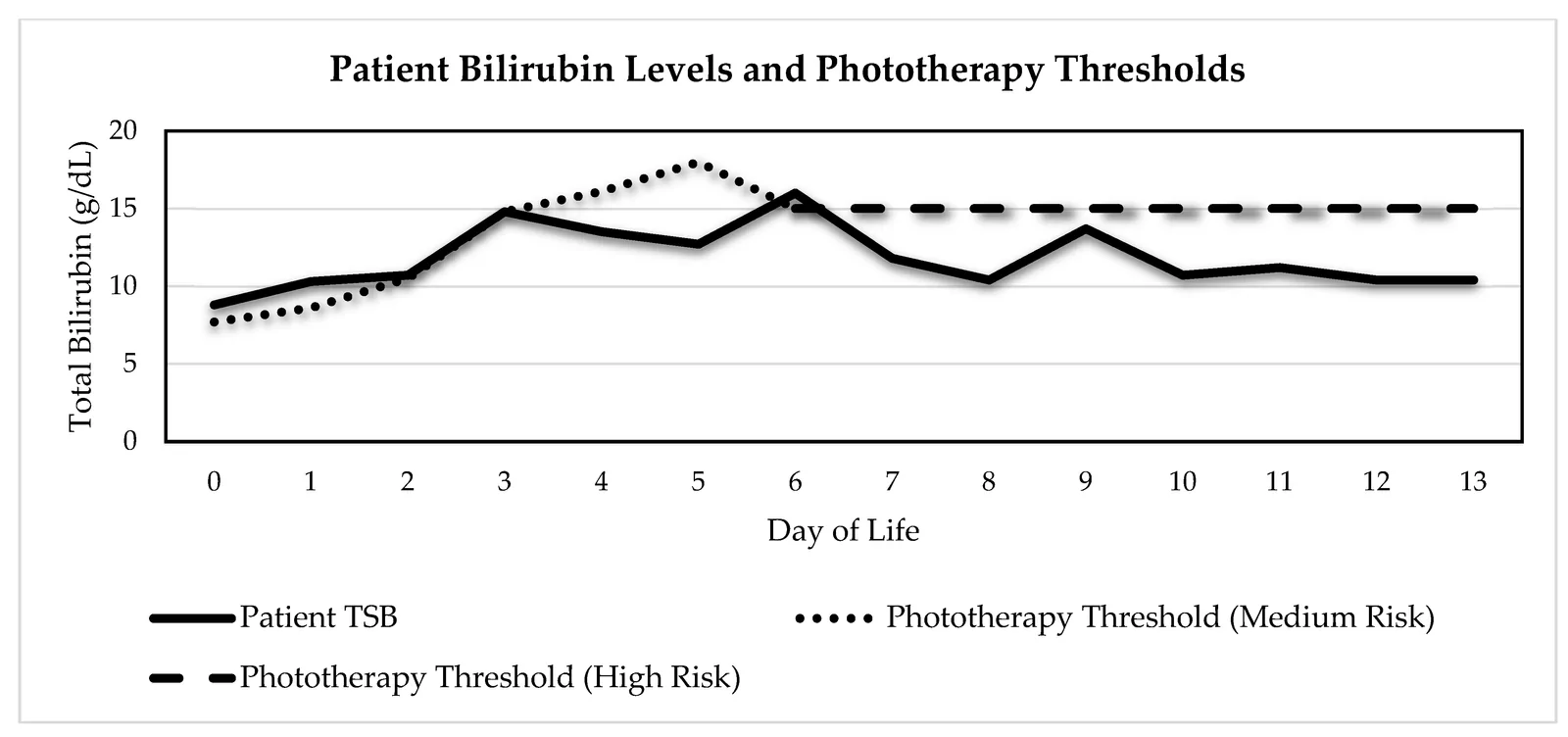
Case I TSB levels and phototherapy thresholds based on 2004 AAP Guidelines in place at the time (Adapted from [6]). Patient was initially plotted on phototherapy nomogram medium-risk line due to gestational age, which was 37w6d. On DOL 6, due to a working diagnosis of DAT-negative ABO HDN, she was plotted on the high-risk line. Her highest total bilirubin was 16 on DOL 6.

The baby had normal physical examinations with no bruising or cephalohematoma noted. She was exclusively breastfed with regular bowel movements and urine output. By our usual NBN practice, she had a transcutaneous total bilirubin (TCTB) at 4 h of life (HOL), which was 4.6, prompting a total serum bilirubin (TSB), which was 5.5 mg/dL at that time. TSB was 8.8 mg/dL at 12 HOL, and she started phototherapy based on the AAP hyperbilirubinemia management guidelines in place at the time (Figure 1) [6]. Her blood type was B− with a negative DAT. During her first week of life, she was on and off phototherapy. TSB was monitored twice daily and peaked on DOL 5 at 16 mg/dL. The NBN team had a working diagnosis of DAT-negative ABO hemolytic disease of the newborn. At DOL 5, they began to consider other etiologies and obtained liver function tests and thyroid function tests, which were normal.

The baby was transferred to the NICU on DOL 8–9. Her hematocrit (HCT) had fallen to 28.8%; therefore, further evaluation for hemolytic causes was performed. Glucose-6-phosphate dehydrogenase deficiency (G6PDd) qualitative screen was normal, and quantitative enzyme assay was 616 U/10^12^ RBC (normal adult reference range 260–728 U/10^12^ RBC). On DOL 9, the neonatologist noted that the mean corpuscular hemoglobin concentration (MCHC) was elevated at 40.6 g/dL and the MCHC:MCV ratio (HS Index) was 0.44 (Table 1), both suggestive of HS [7]. The red cell distribution width (RDW) was 15% (normal 11.5–14.5%). Eosin 5’ maleimide (EMA) testing was performed and was positive with a low mean fluorescent intensity (MFI) of 134 (normal 155–175 MFI), confirming HS.

During the diagnostic work-up, the infant had no further need for phototherapy. She continued to breastfeed well. Her HCT fell to 25.7%, and in consultation with the Pediatric Hematology team, she was given erythropoietin (EPO) at 500 U/kg/dose one day prior to and on the day of discharge with a scheduled follow-up with Pediatric Hematology.

### 2.2. Case II

A 2320 g female infant was born to a 31-year-old African American woman with diet-controlled gestational diabetes at 34w3d gestation via repeat c-section due to non-reassuring non-stress test and severe preeclampsia treated with magnesium infusion. The maternal blood type was A+ antibody negative. PNSs were negative, but GBS was pending at the time of delivery. The mother had a chlamydia infection during pregnancy that was treated with a subsequent negative test of cure.

At delivery, the infant required bag-mask ventilation and transitioned to CPAP +6 for transfer to the NICU. In the NICU, her FiO_2_ decreased from 60 to 27%, and she received surfactant by less invasive surfactant administration (thin catheter with direct laryngoscopy). Her initial blood gas at 1 HOL had an incidental whole blood total bilirubin (GEM TB) of 2.7 mg/dL, and she started phototherapy based on a unit-specific hyperbilirubinemia treatment guideline for babies < 35 weeks gestation. Subsequent GEM TB levels were 4.1 and 5.7 mg/dL at 6 and 12 HOL, respectively, and phototherapy irradiance was increased. Admission complete blood count (CBC) showed an HCT value of 35%, RDW of 16.3%, MCV of 95 fL, and MCHC of 37.7 g/dL, giving an MCHC:MCV ratio of 0.397. The reticulocyte count was 9%. The peripheral smear showed basophilic stippling. Due to the early hyperbilirubinemia and CBC indices, the EMA test was sent on the day of birth and was positive for HS with a patient MFI ratio of 0.59 (normal > 0.9). She received phototherapy for a total of 14 days and received a packed red blood cell transfusion on DOL 13 for HCT 24.2%. She was discharged home on DOL 16.

### 2.3. Case III

A 2870 g full-term white female infant was born by c-section for non-reassuring fetal heart tones. The mother was primigravid with blood type O+ antibody negative, and other PNS were negative. At about 1 HOL, the Labor and Delivery nursing staff called the pediatrician due to the baby appearing jaundiced. TCTB was 6.9 at that time. Stat TSB was sent, and baby was transferred to the NICU for prompt intensive phototherapy. The 1 HOL TSB yielded a result of 7.1 mg/dL. The initial Hct was 38.9%; the reticulocyte count was 12.9%; RDW was 22.7%; MCHC was 36 g/dL; MCV was 112 fL; MCHC:MCV ratio was 0.32; and the peripheral smear showed mild polychromasia and few RBC fragments, spherocytes, target cells, ovalocytes, teardrop cells, and burr cells. Because of very early hyperbilirubinemia and initial reticulocytosis, a hemolytic process was presumed. The baby’s blood type was A+ DAT-negative, so a non-immune etiology was sought. The G6PDd qualitative screen was normal, and the quantitative enzyme level was sent and subsequently found to not be deficient (the test was sent outand results were not available by the time of discharge, and subsequently, the test returned a value of 24.8 U/g Hgb with adult reference range of 9.9–16.6 U/g Hgb). The peak reticulocyte count was 16.5% on DOL 1. Pediatric Hematology was consulted and recommended EMA testing. EMA was positive for HS with an MFI ratio of 0.55 (reference ranges < 0.83 consistent with HS, 0.84–0.9 indeterminate, >0.9 not consistent with HS). The baby was treated with phototherapy in the NICU from 2 to 50 HOL and was discharged to home on DOL 4. On the date of discharge, Hct was 33.5%, the RDW was 17.8%, and the MCHC was 36.7 g/dL with an MCHC:MCV ratio of 0.34.

## 3. Discussion

**Figure 2:**
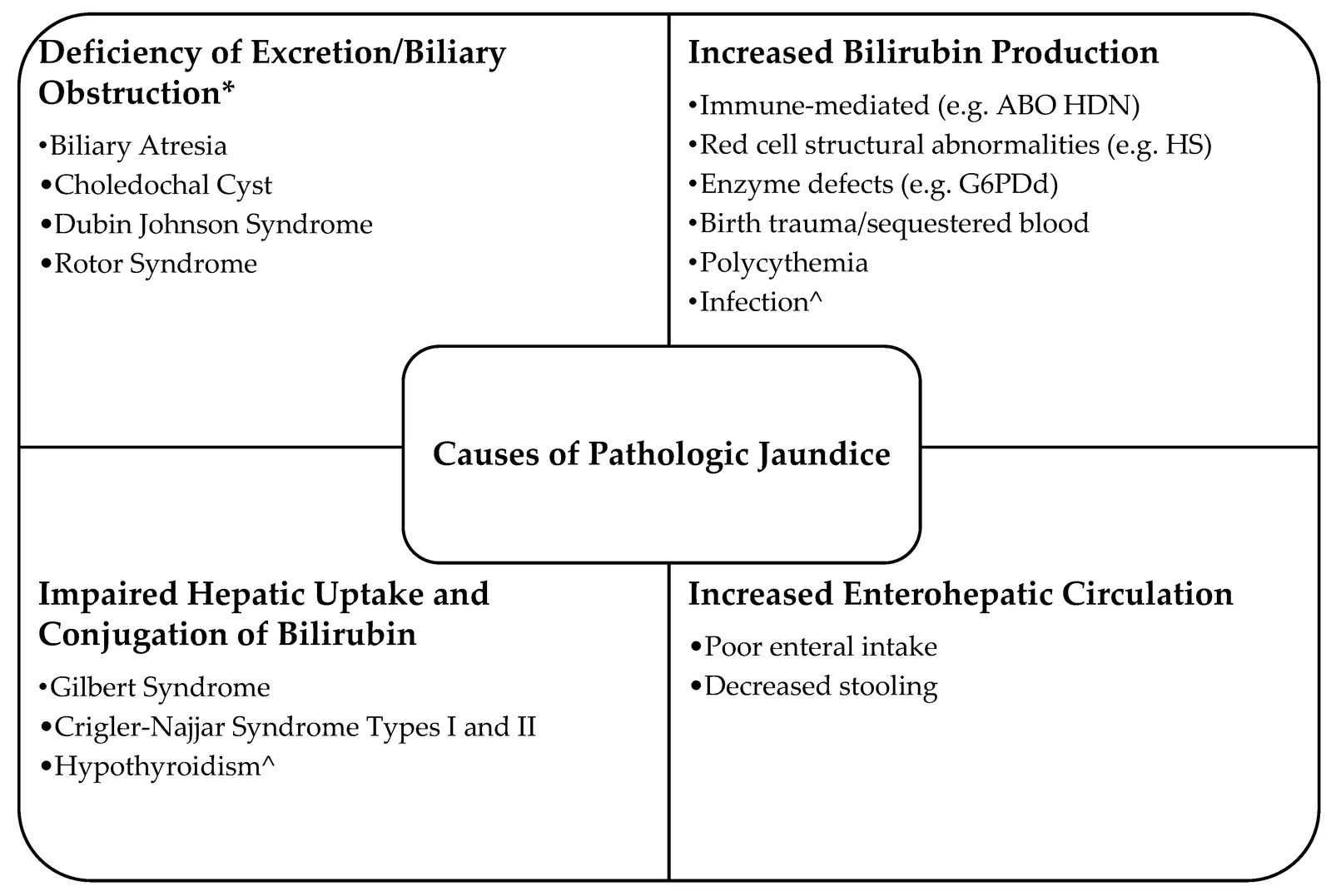
Causes of pathological jaundice (Adapted from [1,2]). * An elevated direct bilirubin is typically seen in disorders related to deficiency of biliary excretion. ^ These etiologies may have more than one of the four mechanisms causing pathologic jaundice.

The various mechanisms of pathologic jaundice can be broken down into four categories: deficiency of excretion/biliary obstruction, increased bilirubin production, impaired conjugation of bilirubin, and increased enterohepatic circulation (Figure 2) [1,2]. Jaundice occurring in the first 24 h of age should prompt a high suspicion for a hemolytic process as most newborns have baseline physiologic immaturity of conjugation, so increased bilirubin production from the shortening of the RBC lifespan, or hemolysis, is often the cause of early and significant hyperbilirubinemia [4,8]. Hemolysis from any cause is delineated as a risk factor for the development of significant neonatal hyperbilirubinemia, and importantly, any hemolytic condition is considered a risk factor for neurotoxicity of bilirubin [5]. Thus, presence of a hemolytic condition as the cause of hyperbilirubinemia prompts lower thresholds for treatments [5]. In addition, the etiology of hemolysis should be sought in order to identify newborns in whom IVIG treatment could be considered [5,9] and those with disorders that have life-long health implications [3]. In each of the presented cases, hemolysis was suspected due to the early hyperbilirubinemia requiring phototherapy, and evaluations for etiology of hemolysis were performed. Below, we discuss etiologies considered. Though HS may not typically be on top of the differential diagnosis list, with these cases and the relative ease of diagnostic evaluation, we recommend that it be taken into consideration when early hyperbilirubinemia from hemolysis is suspected.

### 3.1. DAT-Negative ABO Hemolytic Disease of the Newborn—A Cautionary Tale

A frequent cause of hemolytic jaundice presenting in the first 24 h of age is alloimmune HDN indicated by ABO or Rh mismatch between mother and baby and a positive DAT in the baby. With the regular, widespread use of Rh immunoglobulin, ABO HDN, mostly type A or B infants born to type O mothers, is the most common cause of HDN [10]. Case I and III patients were at risk for ABO HDN due to blood type mismatch between the O mothers and the type B and A babies, respectively. However, both babies had negative DATs. For case I, the NBN team initially attributed her early and prolonged jaundice to DAT-negative ABO HDN. DAT-positive neonates may not have significant hemolysis and hyperbilirubinemia, while DAT-negative neonates can have significant hemolysis from non-immune-mediated etiologies [4,10,11]. Watchko suggests the following: “Absent a positive DAT, the diagnosis of ABO HDN is suspect” [11].

Using ETCOc levels to detect hemolysis, there was no significant difference at 12 HOL between DAT-negative ABO-incompatible newborns and DAT-negative ABO-compatible newborns [12]. Twelve-hour ETCOc levels of DAT-positive ABO-incompatible newborns were significantly higher than in DAT-negative ABO-incompatible newborns, and DAT-positive newborns had a higher rate of significant jaundice than newborns who were DAT-negative [12]. In this study, two DAT-negative ABO-incompatible neonates with elevated ETCOc levels were found to have other etiologies of hemolysis: G6PDd and hereditary elliptocytosis [12]. Similarly, in a report of neonates with acute BE, there were two DAT-negative ABO-incompatible infants with TSB > 25 mg/dL found to have HS and G6PDd [11]. In addition, when analyzing a change from using the AAP medium-risk phototherapy nomogram to the low-risk nomogram [6] for DAT-negative ABO-incompatible newborns, Gabbay et al. concluded that these newborns are at low risk for hyperbilirubinemia requiring phototherapy [13].

ABO HDN should not be the presumed cause of hemolysis and hyperbilirubinemia in ABO-incompatible neonates with a negative DAT. As per Watchko, “Instead, a negative DAT in a severely hyperbilirubinemic ABO incompatible neonate should trigger an exhaustive search for an alternative cause” [11].

### 3.2. Glucose-6-Phosphate Dehydrogenase Deficiency-Related Hemolysis and Hyperbilirubinemia

Glucose-6-phosphate dehydrogenase deficiency (G6PDd) is a common X-linked recessive inherited disorder that may lead to red blood cell hemolysis and increased bilirubin load [14]. Infants with G6PDd are at an increased risk of severe newborn hyperbilirubinemia [15]. Areas of the world with traditionally high incidence include sub-Saharan Africa, the Mediterranean region, the Middle East, and Asia [14]. Some countries with high prevalence have routine newborn screening for G6PDd [14]. Modern migration, ease of travel, and globalization means that G6PDd cannot be considered limited to certain countries or populations [14]. In 2006, testing of United States Army personnel showed 12.2% African American males, 4.3% Asian American males, and 4.1% of African American females to have G6PDd [16]. In the U.S., few states screen for G6PDd in their state newborn screening programs.

Having recognized the risk of G6PDd for severe hyperbilirubinemia, we have a long-standing screening program in male newborns in our newborn nurseries [17] and our NICU [18]. However, our case patients are all females; females are not screened in our program due to the test that is available in-house, which gives rapid turnaround time but may not detect deficiency in female heterozygotes [17]. We recognize this limitation to our program as G6PD-deficient females may develop hyperbilirubinemia as deficient homozygotes or as hemizygotes with unequal Lyonization [19]. So, when considering a hemolytic cause for hyperbilirubinemia in female infants, we will often send both our usual qualitative screen, which will detect the severe deficiency of a homozygous female [17,20] and a quantitative enzyme assay to try to detect deficient heterozygotes.

Universal newborn screening for G6PDd in the U.S. has been debated by various stakeholders over the years. These experts favor universal screening over targeted screening, tests that give results before birth hospitalization discharge to allow for parental counseling, and tests that can identify all newborns at risk for G6PDd-related hemolysis and hyperbilirubinemia: hemizygous males, homozygous females, and heterozygous-affected females [20]. After a New York state mandate to screen infants who are at high risk for G6PDd, Milburn et al. implemented a health system-wide universal screening program, with a test turnaround time of 1.5 days, and determined infant G6PD enzyme level reference ranges since adult reference ranges are typically used [21].

### 3.3. Hereditary Spherocytosis

HS is one of the congenital hemolytic anemias due to RBC membrane or cytoskeleton structural defects or “membranopathies” (Figure 1) [22]. HS is the most common membranopathy and is the third most common hemolytic condition in newborns after ABO isoimmunization and G6PDd [23]. It is also the most common cause of non-immune hemolytic hyperbilirubinemia in neonates with kernicterus [24]. HS is a genetic disorder affecting erythrocyte membrane proteins, most commonly ankyrin-1, band 3, β-spectrin, α-spectrin, and Protein 4.2 [7,25]. Abnormalities of these structural proteins cause spherical, hyperdense, poorly deformable RBC that have a shortened lifespan and are destroyed in the spleen [7,24]. HS occurs in 1 to 2000 white neonates of Northern European ancestry but can be seen in all races and ethnicities [7]. Like G6PDd, because of today’s global world, we should not discount the possibility of HS based on race or ethnicity. A thorough family history is important, and one should not just inquire about an HS diagnosis but also about jaundice, early gallstones, splenectomy, anemia, and blood transfusions [22]. HS is most often inherited in an autosomal dominant pattern, with a parent known to have HS in about 65% of affected neonates [7]. However, up to 30% of individuals with HS have recessive inheritance or de novo mutations; one should not overlook HS because of negative family history [26].

The classic triad associated with HS, anemia, splenomegaly, and jaundice, is rarely seen in neonates. Neonatal presentation ranges from hydrops fetalis to asymptomatic [7,26]. The most common presentation of HS in neonates is jaundice [7,23,26]. Splenomegaly, typically seen in older children and adults with HS, is rarely seen in neonates [7,26]. Spherocytes are a hallmark of the disorder; however, they are not observed in about one-third of affected neonates’ blood smears [7,22,26]. The presence of spherocytes does not always mean HS, as they can be seen in DAT+ ABO incompatibility, although if persistently seen in a hyperbilirubinemic ABO-incompatible DAT-negative neonate, HS should be strongly considered [22].

Complete blood counts (CBCs) are often performed for neonates with significant hyperbilirubinemia. A CBC is recommended by the AAP for infants reaching their escalation of care TSB level, which is 2 mg/dL below their exchange transfusion level [5]. Parameters from the CBC have been shown to be indicative of HS. As spherocytes have lost their biconcave disc shape and central pallor, their MCV is low owing to the loss of a portion of membrane, and the MCHC is elevated [7,23,25,26]. An MCHC > 35.5 g/dL is pathognomonic for spherocytes in older children and adults [25], while an MCHC > 36.5–37 g/dL in neonates indicates that HS is likely [7]. With a detailed study of RBC indices, Delhommeau et al. found an MCHC > 33.5 g/dL in 83% of HS-affected neonates, whereas all age-matched normal newborns had an MCHC below this level [27]. The infants in cases I and II both had an MCHC exceeding 36.5 g/dL. In case III, the patient did not have an MCHC > 36.5 g/dL initially, but it was 36.7 g/dL at discharge on DOL 4.

The MCHC:MCV ratio, also termed “The HS Index,” of >0.36 was shown by Christensen et al. to indicate HS with 97% sensitivity, >99% specificity, and >99% negative predictive value [7]. With concern that Christensen’s work was in a homogeneous, mostly white population, and that MCV decreases from birth to the first 3 months of life, Weiss et al. studied the HS Index in a more diverse population. They found HS in 1.2 per 10,000 infants, with the HS Index being 56% sensitive and 93% specific [28]. Interestingly, in the case patients, the only one without an HS Index > 0.36 during their birth hospitalization was the patient who identified as white; however, she also had the shortest stay of our cases, so her MCV may not have decreased by discharge.

The RDW is a measure of RBC anisocytosis. Increased RDW, or wide variation in RBC size, has been identified as an indicator of HS. Weiss et al. found a considerable difference in the mean RDW between neonates with HS (20%) vs. those without (16.9%) [28]. They concluded that the RDW had better discrimination of HS than the HS Index [28]. Delhommeau et al. found an overlap of RDW results between affected and non-affected neonates; however, the RDW range was 15–25% in the neonates with HS, while those without HS did not have an RDW > 20.5% [27]. The RDW was elevated in all of the presented cases.

While anemia and reticulocytosis are often considered hallmarks of hemolytic conditions, they may not be seen initially in babies with HS. Newborns with HS were found to have similar hemoglobin levels to normal newborns at birth and in the first few DOL [27]. However, after the first week of life, hemoglobin can precipitously drop and lead to pallor, respiratory distress, and/or feeding difficulties [7,26,27]. At birth, like normal newborns, babies with HS often have an elevated reticulocyte count that drops and remains low in subsequent weeks and months owing to robust fetal erythropoiesis being shut down by decreased erythropoietin secretion [27]. Thus, infants with HS who develop significant anemia after the first weeks of life often cannot mount an appropriate erythropoietic response [27]. Delhommeau et al. showed an inverse relationship between reticulocyte count and PRBC transfusion over the first year of life, with most HS infants reaching a reticulocyte count of 200 × 10^9^/L and no longer needing transfusion by one year of age [27].

Osmotic fragility testing is the traditional test for HS. This test evaluates the hemolysis of RBC in varying sodium chloride concentrations with spherocytes showing more than normal hemolysis as the concentration is increased [7]. The osmotic fragility test can be difficult to interpret in neonates as it cannot discriminate between spherocytes present due to HS or other hemolytic conditions [26]. In addition, neonatal RBCs have increased osmotic fragility compared to adult erythrocytes, so this testing is often delayed until after the neonatal period [24,26]. The EMA test emerged in 2000 [29] and is now the preferred method of diagnosing HS [7,24]. It measures fluorescent EMA dye binding to RBC band 3 and other membrane proteins by flow cytometry [7,24]. RBCs in HS exhibit reduced EMA binding [7,24]. Comparing normal newborns to newborns with HS, Christiansen et al. showed no overlap in the ranges of EMA mean fluorescence intensity, so it can be used successfully in neonates [24]. Osmotic gradient ektacytometry is the best test to detect spherocytosis by determining the exact osmotic fragility of the RBC and can differentiate spherocytes from other abnormally shaped cells [27,29]. However, ektacytometry is not typically readily available to clinicians [26,29].

Including HS on the differential diagnosis of hemolytic hyperbilirubinemia and early diagnosis of HS is important, first to understand the etiology of the hyperbilirubinemia to plan appropriate treatment methods and treatment thresholds, and then to provide counseling to families and close follow-up [7,27]. As previously stated, infants with HS often have significant anemia in the first weeks to months of life for which the sluggish erythropoiesis of neonates and infants cannot compensate [27]. Up to 80% receive RBC transfusions in the first year of life [7,27,30]. In addition, patients with a decreased RBC lifespan can have aplastic crisis and severe anemia with infections, particularly with parvovirus [31]. Usually, after the first year of life, the need for transfusion decreases; however, some patients may go on to need splenectomy for chronic anemia, splenomegaly, aplastic crisis, and gallstones [7,27,29].

While RBC transfusion remains the mainstay of treatment for HS-associated anemia, the patient in case I received EPO treatment prior to discharge. EPO has been successfully used for anemia with slow erythropoietic compensation in neonates and infants from other etiologies such as anemia of prematurity in attempt to try to avoid transfusions [23]. EPO stimulates the production of RBC and has shown promise for the treatment of HS-related anemia [7,23,32]. Tchernia et al. used EPO in 16 infants with HS at a mean of 45 DOL. Eighty-one percent of these infants were either good responders (no PRBC transfusions once EPO started) or partial responders (one PRBC transfusion during EPO treatment with none after EPO discontinued) [30]. An Italian multicenter trial showed that infants treated with EPO ± PRBC transfusions received significantly less transfusions than those treated with PRBC transfusion alone [32]. In this study, high vs. low doses of EPO did not give a difference in outcomes, there were no noted side effects from EPO treatment, the mean treatment duration was 19 weeks, and the mean number of injections given per week was two [32]. The authors recommend starting EPO as soon as possible after birth in infants with HS and early anemia as the reticulocyte count in the EPO-treated group was higher at 30 DOL than in the transfusion only group, whereas it was similar between the two groups at 180 DOL [32]. Despite these results, the cost of EPO relative to transfusion and issues with EPO administration in the outpatient setting, particularly the need for injections multiple times per week, have been cited as reasons that EPO has not become the standard treatment for HS-associated anemia at this time [32].

## 4. Conclusions

It is important to determine whether there is a hemolytic cause for severe neonatal hyperbilirubinemia as treatment should begin at a lower TSB threshold when hemolytic conditions are present and hemolysis is a risk for the neurotoxicity of bilirubin [5]. However, it is also important to determine the underlying cause of hemolysis when present in neonates, as inherent RBC disorders have implications for long-term health. Hemolysis-related pathologic jaundice should not be attributed to ABO HDN in the absence of a positive DAT, and an expanded differential diagnosis should be considered. All of the cases presented had a negative family history for HS and were of varied backgrounds. A negative family history or patient race/ethnicity should not be a deterrent in including HS in the differential diagnosis. Attention to CBC parameters—a test often performed for neonates with hyperbilirubinemia—including the MCHC, MCHC:MCV ratio, and RDW can be helpful in evaluating a patient for HS and directing clinicians to perform more definitive diagnostic testing [26].

## Figures and Tables

**Table 1:** Hematologic values during the birth hospitalization course—Case I.

Timing of Lab Draw, Patient Location	HCT (%)	Reticulocyte Count (%)	MCHC * (g/dL)	MCV (fL)	MCHC:MCV *	Pathologist Peripheral Smear Report
HOL11, NBN	40.7	7.4	36.1	108	0.33	n/a
DOL9, NICU	28.8	1.7	40.6	93	0.44	Mild polychromasia
DOL11, NICU	25.7	2.2	37.7	97	0.39	Mild polychromasia ^

HOL = hour of life, DOL = day of life, NBN = newborn nursery, NICU = neonatal intensive care unit, HCT = hematocrit, MCHC = mean corpuscular hemoglobin concentration, MCV = mean corpuscular volume. * MCHC ≥ *36.5–37 g/dL and MCHC:MCV ratio of >0.36 highly suggestive of hereditary spherocytosis (Adapted from* [7]). *^ pediatric hematologist reviewed smear and identified spherocytes.*

## Data Availability

The original contributions presented in this study are included in the article. Further inquiries can be directed to the corresponding author.

## Abbreviations

The following abbreviations are used in this manuscript

ABO	Blood Types A, B, and O
AAP	American Academy of Pediatrics
BE	Bilirubin Encephalopathy
CBC	Complete Blood Count
DAT	Direct Antiglobulin Test
DOL	Day(s) of Life
EMA	Eosin 5’ Maleimide
ETCOc	End Tidal Carbon Monoxide (corrected for ambient carbon dioxide).
G6PDd	Glucose-6-Phosphate Dehydrogenase Deficiency
GBS	Group B Streptococcus
GEM TB	Whole Blood Total Bilirubin
HCT	Hematocrit
HDN	Hemolytic Disease of the Newborn
HOL	Hour(s) of Life
HS	Hereditary Spherocytosis
IVIG	Intravenous Immunoglobin
MCHC	Mean Corpuscular Hemoglobin Concentration
MCV	Mean Corpuscular Volume
MFI	Mean Fluorescent Intensity
NBN	Newborn Nursery
NICU	Neonatal Intensive Care Unit
PNS	Prenatal Screens
RBC	Red Blood Cell
RDW	Red Cell Distribution Width
TCTB	Transcutaneous Bilirubin
TSB	Total Serum Bilirubin

## References

[1] Ansong-Assoku B., Adnan M., Daley S.F., Ankola P.A. (2025). Neonatal jaundice [Updated 2024 Feb 12]. StatPearls [Internet].

[2] Kaplan M., Wong R.J., Burgis J.C., Sibley E., Stevenson D.K., Fanaroff A.A., Martin R.J., Walsh M.C. (2020). Neonatal jaundice and liver diseases. Fanaroff and Martin’s Neonatal-Perinatal Medicine: Diseases of the Fetus and Infant.

[3] Christensen R.D., Yaish H.M., Lemons R.S. (2014). Neonatal hemolytic jaundice: Morphologic features of erythrocytes that will help you diagnose the underlying condition. Neonatology.

[4] Christensen R.D., Bahr T.M., Wong R.J., Vreman H.J., Bhutani V.K., Stevenson D.K. (2023). A “gold standard” test for diagnosing and quantifying hemolysis in neonates and infants. J. Perinatol..

[5] Kemper A.R., Newman T.B., Slaughter J.L., Maisels M.J., Watchko J.F., Downs S.M., Grout R.W., Bundy D.G., Stark A.R., Bogen D.L. (2022). Clinical practice guideline revision: Management of hyperbilirubinemia in the Newborn infant 35 or more weeks of gestation. Pediatrics.

[6] American Academy of Pediatrics Subcommittee on Hyperbilirubinemia (2004). Management of hyperbilirubinemia in the newborn infant 35 or more weeks of gestation. Pediatrics.

[7] Christensen R.D., Yaish H.M., Gallagher P.G. (2015). A pediatrician’s practical guide to diagnosing and treating hereditary spherocytosis in neonates. Pediatrics.

[8] Wong R.J., Bhutani V.K., Stevenson D.K. (2017). The importance of hemolysis and its clinical detection in neonates with hyperbilirubinemia. Curr. Pediatr. Rev..

[9] Slaughter J.L., Kemper A.R., Newman T.B. (2022). Technical report: Management of hyperbilirubinemia in the Newborn infant 35 or more weeks of gestation. Pediatrics.

[10] Christensen R.D., Bahr T.M., Ilstrup S.J., Dizon-Towson D.S. (2023). Alloimmune hemolytic disease of the fetus and newborn: Genetics, structure, and function of the commonly involved erythrocyte antigens. J. Perinatol..

[11] Watchko J.F. (2023). ABO hemolytic disease of the newborn: A need for clarity and consistency in diagnosis. J. Perinatol..

[12] Herschel M., Karrison T., Wen M., Caldarelli L., Baron B. (2002). Isoimmunization is unlikely to be the cause of hemolysis in ABO-incompatible but direct antiglobulin test-negative neonates. Pediatrics.

[13] Gabbay J.M., Agneta E.M., Turkington S., Bajaj B.M., Sinha B., Geha T. (2023). Rates of phototherapy among ABO-incompatible newborns with a negative direct antiglobulin test. J. Perinatol..

[14] Kaplan M., Hammerman C. (2009). The need for neonatal glucose-6-phosphate-dehydrogenase screening: A global perspective. J. Perinatol..

[15] Kaplan M., Hammerman C., Bhutani V.K. (2015). Parental education and the WHO neonatal G-6-PD screening program: A quarter century later. J. Perinatol..

[16] Chinevere T.D., Murray C.K., Grant E., Johnson G.A., Duelm F., Hospenthal D.R. (2006). Prevalence of glucose-6-phosphate dehydrogenase deficiency in U.S. Army personnel. Mil. Med..

[17] Nock M.L., Johnson E.M., Krugman R.R., Di Fiore J.M., Fitzgerald S., Sandhaus L., Walsh M.C. (2011). Implementation and analysis of a pilot in-hospital newborn screening program for glucose-6-phosphate dehydrogenase deficiency in the United States. J. Perinatol..

[18] Lam R., Li H., Nock M.L. (2015). Assessment of G6PD screening program in premature infants in a NICU. J. Perinatol..

[19] Kaplan M., Beutler E., Vreman H.J., Hammerman C., Levy-Lahad E., Renbaum P., Stevenson D.K. (1999). Neonatal hyperbilirubinemia in glucse-6-phospahte dehydrogenase-deficient heterozygotes. Pediatrics.

[20] Watchko J.F., Kaplan M., Stevenson D.K., Bhutani V.K. (2013). Should we screen newborns for glucose-6-phosphate dehydrogenase deficiency in the United States?. J. Perinatol..

[21] Milburn S., Bhutani V.K., Weintraub A., Guttmann K. (2024). Implementation of universal screening for G6PD deficiency in newborns. Pediatrics.

[22] Cortesi V., Manzoni F., Raffaeli G., Cavallaro G., Fattizzo B., Amelio G.S., Silvia G., Amodeo I., Giannotta J.A., Mosca F. (2021). Severe presentation of congenital hemolytic anemias in the neonatal age: Diagnostic and therapeutic issues. Diagnostics.

[23] Coramusi C., Lucangeli N., Vadala S., Parisi P., Scapillati M.E. (2025). Neonatal hereditary spherocytosis: A case report. Ital J Pediatr.

[24] Christensen R.D., Agarwal A.M., Nussenzveig R.H., Heikal N., Liew M.A., Yaish H.M. (2015). Evaluating eosin-5-maleimide binding as a diagnostic test for hereditary spherocytosis in newborn infants. J. Perinatol..

[25] Christensen R.D., Henry E. (2010). Hereditary spherocytosis in neonates with hyperbilirubinemia. Pediatrics.

[26] Gallagher P.G. (2021). Difficulty in diagnosis of hereditary spherocytosis in the neonate. Pediatrics.

[27] Delhommeau F., Cynober T., Schischmanoff P.-O., Rohrlich P., Delaunay J., Mohandas N., Tchernia G. (2000). Natural history of hereditary spherocytosis during the first year of life. Blood.

[28] Weiss N.M., Kuzniewicz M.W., Shimano K.A., Walsh E.M., Newman T.B. (2021). Use of complete blood cell count components to screen for hereditary spherocytosis in neonates. Pediatrics.

[29] Manciu S., Matei E., Trandafir B. (2017). Hereditary spherocytosis—Diagnosis, surgical treatment and outcomes. A literature review. Chirurgia.

[30] Tchernia G., Delhommeau F., Perrotta S., Cynober T., Bader-Meunier B., Nobili B., Rohrlich P., Salomon J.L., Sagot-Bevenot S., Miraglia del Gidice E. (2000). Recombinant erythropoietin therapy as an alternative to blood transfusions in infants with hereditary spherocytosis. Hematol. J..

[31] Steward S.C., Chauvenet A.R., O’Suoji C. (2014). Hereditary spherocytosis: Consequences of delayed diagnosis. SAGE Open Med..

[32] Farruggia P., Puccio G., Ramenghi U., Colombatti R., Corti P., Trizzino A., Barone A., Boscarol G., Ferraro F., Grotto P. (2017). Recombinant erythropoietin vs. blood transfusion care in infants with hereditary spherocytosis: A retrospective cohort study of A.I.E.O.P. patients (Associazione Italiana Emato-Oncologia Pediatrica). Am. J. Hematol..

